# Women in Industry

**Published:** 1921-03-05

**Authors:** 


### Women in Industry.

Dr. Louise McIlroy, whose appointment to the directorship of the Obstetrical and Gynæcological Unit of the Royal Free Hospital and London School of Medicine for Women we announced last week, has already entered upon her new responsibilities. One of the vital problems which are to be studied in the research department, of which she is now the head, is evidently to be that of the effect of women's various employments upon their health and on that of their children. In an interview Dr. McIlroy declared that this is a matter in which there should be the fullest inquiry, and one in which trained women should be able to give important help. There is, without doubt, she says, going to be a big battle on the question of the suitability of women, and more especially of married women, for all sorts of work. The problem will have to be faced, and it is very desirable that a mass of records should be collected and scientifically studied, so that an authoritative pronouncement may be made.

"We have got far away," she added, "from the days when it was thought proper that women should be ailing, delicate creatures. Events have shown what good work the normal woman can do, but the idea still persists that they are subjected to singular disabilities which depreciate their economic value. My idea is that women should be equal with men in every way, and that individual cases of ill-health should not characterise the whole sex as physically weak any more than do individual cases of ill-health among men. The normal woman is well fitted to take her place in the industrial world." That is a bolder view than we are accustomed to hear from experts who are inclined to ignore the surprising capacity for endurance and hard work which women displayed under the special conditions brought about by the war. The explanation in general terms of the success with which, during the war period, women, large numbers of whom hitherto had no experience of industrial labour, worked in the factories is not difficult. In the first place, it is often forgotten that housework is more exhausting than ordinary work, and that the mother's surroundings in the modern factory are probably much healthier than her home surroundings—an assumption certainly justified in the case of many large war factories in which the health of the women was carefully supervised, and in which the conditions of work were adjusted with a good deal of administrative skill. Dr. McIlroy even goes so far as to say that the expectant mother who is in need of her earnings should be allowed to go on working, though gradually the work should be modified, while after the birth of her child provision should be made to enable her to look after it. There is undoubtedly a good deal of prejudice on the part of some employers against married women—even the Ministry of Health has a rule, we believe, that women in its employ must resign when they get married—and there is also a genuine sentiment on the part of many social workers against married-women employment. But in the changed conditions of economic life there is no reason why a woman, merely because she is married, should be barred from employment if the limits of her capacity can be scientifically defined, and the nature of her work accommodated to her physique and rendered innocuous to her maternal functions.

